# Novel role for epalrestat: protecting against NLRP3 inflammasome-driven NASH by targeting aldose reductase

**DOI:** 10.1186/s12967-023-04380-4

**Published:** 2023-10-07

**Authors:** Wei Shi, Guang Xu, Yuan Gao, Jun Zhao, Tingting Liu, Jia Zhao, Huijie Yang, Ziying Wei, Hui Li, An-Long Xu, Zhaofang Bai, Xiaohe Xiao

**Affiliations:** 1 School of Life Sciences, Beijing University of Chinese Medicine, Beijing, China; 2 Department of Hepatology, The Fifth Medical Center of PLA General Hospital, Beijing, China; 3 School of Traditional Chinese Medicine, Capital Medical University, Beijing, China; 4 Military Institute of Chinese Materia, The Fifth Medical Center of PLA General Hospital, Beijing, China; 5 The Third Affiliated Hospital of Zunyi Medical University (The First People’s Hospital of Zunyi), Zunyi, China

**Keywords:** Epalrestat, NASH, Aldose reductase, NLRP3 inflammasome, NLRP3 inflammasome-driven disease

## Abstract

**Background:**

Nonalcoholic steatohepatitis (NASH) is a progressive and inflammatory subtype of nonalcoholic fatty liver disease (NAFLD) characterized by hepatocellular injury, inflammation, and fibrosis in various stages. More than 20% of patients with NASH will progress to cirrhosis. Currently, there is a lack of clinically effective drugs for treating NASH, as improving liver histology in NASH is difficult to achieve and maintain through weight loss alone. Hence, the present study aimed to investigate potential therapeutic drugs for NASH.

**Methods:**

BMDMs and THP1 cells were used to construct an inflammasome activation model, and then we evaluated the effect of epalrestat on the NLRP3 inflammasome activation. Western blot, real-time qPCR, flow cytometry, and ELISA were used to evaluate the mechanism of epalrestat on NLRP3 inflammasome activation. Next, MCD-induced NASH models were used to evaluate the therapeutic effects of epalrestat in vivo. In addition, to evaluate the safety of epalrestat in vivo, mice were gavaged with epalrestat daily for 14 days.

**Results:**

Epalrestat, a clinically effective and safe drug, inhibits NLRP3 inflammasome activation by acting upstream of caspase-1 and inducing ASC oligomerization. Importantly, epalrestat exerts its inhibitory effect on NLRP3 inflammasome activation by inhibiting the activation of aldose reductase. Further investigation revealed that the administration of epalrestat inhibited NLRP3 inflammasome activation in *vivo*, alleviating liver inflammation and improving NASH pathology.

**Conclusions:**

Our study indicated that epalrestat, an aldose reductase inhibitor, effectively suppressed NLRP3 inflammasome activation in *vivo* and in *vitro* and might be a new therapeutic approach for NASH.

**Graphical Abstract:**

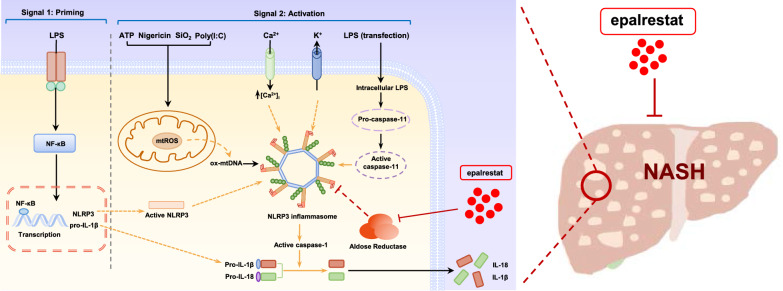

**Supplementary Information:**

The online version contains supplementary material available at 10.1186/s12967-023-04380-4.

## Introduction

Nonalcoholic fatty liver disease (NAFLD) is part of a spectrum of liver diseases called hepatic steatosis and one of the most common liver diseases. It is characterized by the accumulation of large blisters of triglycerides in hepatocytes and occurs in the absence of secondary causes [1, 2]. Nonalcoholic steatohepatitis (NASH) is a progressive and inflammatory subtype of NAFLD characterized by hepatocellular injury and inflammation, with various stages of fibrosis [3, 4]. A small percentage of patients with simple steatosis will progress to cirrhosis, but more than 20% of patients with NASH will.[1, 5] Patients with NASH have a significantly higher risk of developing hepatocellular carcinoma than those with other diseases [6]. According to a survey, from 2004 to 2016, there was a 114% and 80% increase in the number of men and women, respectively, in the liver transplant waitlist for NASH [7]. At the same time, patients with NASH are more susceptible to developing cirrhosis, end-stage liver disease, and an increased risk of morbidity and mortality, even if the disease is often clinically silent [8]. As a result, diagnosing and treating NASH has become a formidable challenge for clinicians.

Although several drugs for NASH have entered phase 3 clinical trials in the past few years, no FDA-approved medical treatment for NASH has been identified [9–11]. At present, the therapeutic interventions for NASH focus on weight loss and lifestyle changes, such as diet and exercise. The demand for pharmacological therapy for NASH remains high because the degree of weight loss required for the histological improvement of the liver is difficult to achieve and maintain [12]. Unfortunately, the only effective therapy for end-stage liver disease and liver failure is liver transplantation [12, 13].

Previous studies indicate that multiple factors can predispose a person to the initial development of NASH, including metabolic alterations and genetic susceptibility [14]. However, accumulating evidence has shown that inflammation and inflammatory signaling pathways play an important role in NASH progression [15, 16]. The activation of liver resident macrophages or Kupffer cells induces NLRP3 inflammasome activation, triggering an inflammatory response. NLRP3 inflammasome activation leads to a broad immune response, including the production of pro-inflammatory cytokines and chemokines, the subsequent recruitment of neutrophils and other immune cells, and cell death [17, 18]. Growing evidence has shown that inflammasome-driven inflammation is associated with tissue damage and liver fibrosis in NASH [17–19].

Inflammasomes are cytoplasmic multiprotein complexes usually composed of NLRP3 (NOD-, LRR- and pyrin domain-containing protein (3), the adaptor protein ASC (apoptosis-associated speck-like protein containing CARD), and the effector molecule pro-caspase-1 [20–22]. Upon stimulation, NLRP3 binds to ASC, which in turn interacts with the cysteine protease caspase-1 and forms a complex that leads to caspase-1 activation. Caspase-1 activation can promote cleavage of the pro-inflammatory cytokines interleukin-1β (IL-1β) and IL-18 into their active forms and the release of the cytosolic protein GSDMD [23, 24]. It is important to note that the development of type 2 diabetes, mitochondrial dysfunction, and insulin resistance are strongly associated with NLRP3 inflammasome activation, which are major risk factors for developing NASH [25, 26].

Several compounds, including MCC950, OLT1177, parthenolide, sulforaphane, and isoliquiritigenin, have been shown to strongly inhibit NLRP3 inflammasome activation [27–32]. MCC950 is a well-studied and specific NLRP3 inhibitor, which can alleviate the symptoms of mouse models of NLRP3-dependent disease, including NASH, type 2 diabetes, and Alzheimer’s disease [27]. However, MCC950 could induce potential hepatotoxicity in phase II clinical trials. In addition, OLT1177, a selective inhibitor of the NLRP3 inflammasome, also has been investigated in phase II clinical trials [28, 33]. Therefore, it is important to develop effective, broadly applicable, and safe NLRP3 inflammasome inhibitors to treat inflammasome-mediated diseases.

Epalrestat, an aldose reductase inhibitor, is used to improve the function of the peripheral nerves in diabetes mellitus [34]. Aldose reductase is a rate-controlling enzyme in the polyol pathway and is a potential drug target for preventing and treating diabetic neuropathy [35, 36]. Several key inhibitors of aldose reductase have been developed to treat diabetes complications via the polyol pathway [37, 38]. Nevertheless, epalrestat is the only aldose reductase inhibitor currently available for clinical use in many countries, including China, Japan, and India [39, 40]. Therefore, epalrestat is a clinically effective and safe drug.

In this study, we report that epalrestat prevented the progression of diabetic retinopathy/nephropathy and could also be used as an anti-inflammatory agent. Here, we describe epalrestat as a highly potent and specific inhibitor of NLRP3, which is active in various NLRP3-dependent mouse models, particularly in NASH.

## Materials and methods

### Mice

C57BL/6 mice were obtained from SPF Biotechnology Co., Ltd (Beijing, China). All animals were reared at SPF conditions, with 40–70% humidity and 12 h of light/12 h of dark per day.

### Cell culture

Bone marrow-derived macrophages (BMDMs) were obtained from C57BL/6 mice (10 weeks old) and cultured in Dulbecco’s modified Eagle’s medium (DMEM) supplemented with 10% FBS, 1% penicillin/streptomycin (P/S) and 50 ng/mL murine macrophage colony-stimulating factor (M-CSF). THP1 cells were cultured in RPMI-1640 medium supplemented with 10% FBS and 1% P/S. HEK-293 T cells were cultured in DMEM supplemented with 10% FBS and 1% P/S. All cells were incubated at 37 °C in a humidified atmosphere with 5% (v/v) CO_2_.

### Antibodies and reagents

Phorbol-12-myristate-13-acetate (PMA), dimethyl sulfoxide (DMSO), adenosine triphosphate (ATP), Nigericin, LPS, TRIzol, and ultrapure LPS were purchased from Sigma-Aldrich (Munich, Germany). Silicon dioxide (SiO_2_), poly (I: C), poly (dA: dT), and Pam3CSK4 were obtained from InvivoGen (San Diego, USA). MCC950 was purchased from TopScience (Shanghai, China). Epalrestat, ponalrestat, ranirestat, and tolrestat were purchased from MedChemExpress (New Jersey, USA). MitoSOX was purchased from Invitrogen (Carlsbad, USA). Certified Fetal Bovine Serum (FBS) were obtained from VivaCell (Shanghai, China). Anti-mouse caspase-1 antibodies (AG-20B-0042) were purchased from Adipogen (San Diego, USA). Anti-human cleaved IL-1β (12,242), anti-human caspase-1 (4199S), anti-mouse IL-1β (12,507), and anti-NLRP3 (15101S) antibodies were purchased from CST (Boston, USA). Anti-ASC (sc-22514-R) antibodies were purchased from Santa Cruz Biotechnology (Dallas, USA). Anti-DDDK tag (20,543-1-AP) and anti-GAPDH (60,004-1-1G) antibodies were purchased from the Proteintech Group (Chicago, USA). Anti-β-actin (ab8226), anti-NEK7 (ab133514), and anti-aldose reductase (ab268058) antibodies were purchased from Abcam (Cambridge, UK). Color Prestained Protein marker (20AB01) was purchased from GenStar (Beijing, China). *Salmonella* was a gift from Dr. Tao Li of the National Center of Biomedical Analysis (Beijing, China).

### Plasmids and transfection

The plasmids pCMV-Flag-Vector and pCMV-NLRP3-Flag were kindly provided by Dr. Tao Li from the National Center of Biomedical Analysis (Beijing, China).

### Inflammasome activation

THP1 and BMDMs were seeded in 24-well culture dishes at a density 1 × 10^6^ cells/mL or 1.5 × 10^6^ cells/mL and cultured overnight. Afterward, the cells were primed for 4 h with 50 ng/mL LPS. Then, the medium was replaced with Opti-MEM supplemented with epalrestat. After 1 h, the activation of NLRP3 was typically achieved through the following treatments: 5 mM ATP for 45 min, 7.5 μM nigericin for 30 min, 200 μg/mL SiO_2_ for 6 h, or transfection with 1 μg/mL poly (I: C) using Lipofectamine 2000 for 6 h. NLRC4 or AIM2 inflammasome activation was accomplished using *Salmonella* or transfection with 1 μg/mL poly (dA: dT) using Lipofectamine 2000 for 6 h.

### Western blot analysis

To detect caspase-1 p20 and IL-1β production, the cell supernatants were concentrated with TCA and then centrifuged at 12,000 ×*g* for 10 min at 4 °C. Next, the concentrated proteins were washed by turning up and down with acetone and centrifuged at 12,000 ×*g* for 5 min at 4 °C. The acetone-containing supernatant was discarded, the samples were placed in 1 × loading sample buffer, and the proteins were denatured at 105 ℃ for 15 min. The protein samples were then resolved on SDS–PAGE gels and transferred to a polyvinylidene difluoride (PVDF) membrane using a wet-transfer system. Next, the PVDF membranes were blocked in TBST (20 mM Tris–HCl [pH 7.6], 150 mM NaCl, and 0.1% [v/v] Tween-20) containing 5% (w/v) non-fat milk for 1 h at room temperature and then incubated with primary antibodies diluted in 5% (w/v) BSA in TBST overnight at 4 °C. Afterward, the membranes were washed thrice with TBST and incubated with the corresponding horseradish peroxidase (HRP)-conjugated secondary antibodies diluted in 5% (w/v) non-fat milk in TBST for 1 h. Following three washes with TBST, the bands generated on the membrane were visualized on X-ray film using a chemiluminescent western blotting detection system.

To detect the expression of NLRP3, pro-IL-1β, ASC, and caspase-1 p45, the cell lysates were collected via direct lysis in a 1 × loading sample buffer. Western blot analysis was then performed as described above.

### Cell viability assay

BMDMs were seeded overnight at a density of 1 × 10^6^ cells/mL in 96-well culture dishes. The cells were then incubated at 37 °C and treated with epalrestat for 24 h. After incubation, the spent medium was replaced with DMEM containing a with cell counting kit-8 (CCK-8) reagent and the cells were incubated for 30 min. The optical density (OD) values were then measured at 450 nm.

### Lactate dehydrogenase (LDH) assay

The release of LDH in the cell supernatants was measured using an LDH cytotoxicity assay kit (Beyotime, Shanghai, China) according to the manufacturer’s instructions.

### Enzyme-linked immunosorbent assay (ELISA)

Cell culture supernatants, peritoneal lavage fluid, and mouse serum were collected. ELISA kits to detect mouse IL-1β (R&D Systems, SMLB00C), TNF-α (Dakewe, 1217202), and IL-6 (Dakewe, 1210602), and human IL-1β (Dakewe, 1110122) and TNF-α (Dakewe, 1117202) were used to detect the levels of the indicated cytokines according to the respective manufacturer’s instructions.

### Caspase-1 activity assay

The activity of caspase-1 in the cell supernatants was measured using a Caspase-Glo^®^ 1 Inflammasome Assay (Promega, Beijing, China) according to the manufacturer’s instructions.

### Real-time PCR analysis

Total RNA from mouse tissue was extracted using TRIzol reagent. cDNA was then synthesized from 2 μg RNA using an RT Master Mix for qPCR (MCE, HY-K0510) and analyzed with a SYBR Green qPCR Master Mix (MCE, HY-K0522) using an iQ6 Real-Time PCR Detection System (Bio-Rad) for real-time PCR analysis. The mRNA level of the target genes was normalized to that of the housekeeping gene *GAPDH*.

### ASC oligomerization

BMDMs were seeded in 12-well culture dishes at density of 1 × 10^6^ cells/mL overnight and then treated with 50 ng/mL LPS for 4 h. Next, the spent medium was replaced with Opti-MEM supplemented with epalrestat and incubated for 1 h. NLRP3, NLRC4, and AIM2 inflammasome activation was achieved using similar treatments as described above. Cells were lysed with a Triton buffer [50 mM Tris–HCl (pH 7.5), 150 mM NaCl, 0.5% Triton X-100, and EDTA-free protease inhibitor cocktail] and then centrifuged at 6000 × *g* for 10 min at 4 °C. Supernatants were then centrifuged at 6000 × *g* for 10 min. The pellet fractions were then washed with PBS and cross-linked with 2 mM disuccinimidyl suberate (DSS) in PBS for 30 min at 37 °C, followed by centrifugation at 6000 ×*g* for 15 min. Finally, the cross-linked pellets were dissolved in 1 × loading buffer and analyzed using a chemiluminescent western blotting detection system.

### Immunoprecipitation and pull-down assays

HEK-293 T cells were transfected with Flag-tagged plasmids (Flag-Vector and Flag-NLRP3) for 24 h and then treated with epalrestat for 6 h. Afterward, the cells were lysed with a lysis buffer (50 mM NaCl, 50 mM Tris, pH 7.8, 0.1% [v/v] Nonidet-P40, 5 mM EDTA and 10% [v/v] glycerol) containing an EDTA-free protease inhibitor cocktail and the cell lysates were collected and centrifuged at 12,000 rpm for 15 min. The supernatants were then immunoprecipitated with anti-Flag M2 affinity beads according to the manufacturer’s instructions. Cell lysates or immunoprecipitates were separated using a chemiluminescent western blotting detection system.

Epalrestat was also conjugated with EAH-activated Sepharose 4B (GE Healthcare). RIPA buffer containing an EDTA-free protease inhibitor cocktail was used to lyse BMDMs, and the lysates were centrifuged at 12,000 ×*g* for 15 min at 4 °C. Next, the combination of epalrestat-conjugated Sepharose 4B beads and cell lysates were co-incubated at 4 °C overnight. Finally, the beads were washed six times with RIPA buffer, and the proteins were analyzed via immunoblotting.

### Intracellular K^+^ and Ca^2+^ measurements

BMDMs were seeded in 12-well plates overnight and then treated with 50 ng/mL LPS for 4 h. The spent medium was then replaced with Opti-MEM supplemented with epalrestat. The cells were then incubated for 1 h and then treated with nigericin or ATP. To measure the intracellular K^+^ levels, the spent medium was aspirated before washing the cells three times with PBS. Ultrapure HNO_3_ was then added to lyse the cells, and the lysates were boiled at 100 °C for 30 min. Intracellular K^+^ measurements were then performed via inductively coupled plasma mass spectrometry. To measure the intracellular Ca^2+^ levels, a trace showing ATP-induced Ca^2+^ flux was analyzed using a FLIPRT Tetra system (Molecular Devices, USA).

### Toxicity of epalrestat in vivo

8 week-old male or female C57BL/6 mice were gavaged with epalrestat (120 mg/kg/day) or vehicle daily for 14 days. The body weight of the mice was measured daily. On the 15th day, after anesthetization, the serum of the mice was collected and assessed for AST, ALT, creatinine, TBIL, and glucose levels using commercial kits according to their respective manufacturer’s instructions.

### LPS-induced systemic inflammation

8 week-old female C57BL/6 mice were gavaged with epalrestat (20 or 40 mg/kg), MCC950 (40 mg/kg), or vehicle for 1 h. After intraperitoneal injection with LPS (20 mg/kg) for 6 h, the mice were anesthetized, and the serum and peritoneal lavage fluids were collected. Cytokine levels in the lavage fluids and serum were detected using ELISA. The stained cells were analyzed using flow cytometry.

### Methionine- and choline-deficient (MCD) diet-induced steatohepatitis and fibrosis

C57BL/6 mice (8 week-old, male) were fed with an MCD diet (518,810, Dyets, USA) or an identical diet supplemented with methionine and choline (MCS; 518,811, Dyets) for 6 weeks according to the manufacturer’s instructions. Afterward, the mice were randomly separated into groups and gavaged with epalrestat (20 mg/kg), MCC950 (20 mg/kg), or vehicle daily for a total of 5 days, and then 40 mg/kg every other day, for up to 6 weeks. Finally, the mice were anesthetized, and their liver and serum were collected for analysis.

### Statistical analyses

All statistical calculations were performed using GraphPad Prism 7 software (GraphPad Software) and Microsoft Excel. Data are expressed as the mean ± SD. Statistical analysis was carried out using a standard two-tailed unpaired Student’s* t*-test for single comparisons and one-way ANOVA for multiple comparisons. Data were considered statistically significant when P < 0.05.

## Results

### Epalrestat inhibits NLRP3 inflammasome activation in BMDMs and THP1 cells

**Fig. 1:**
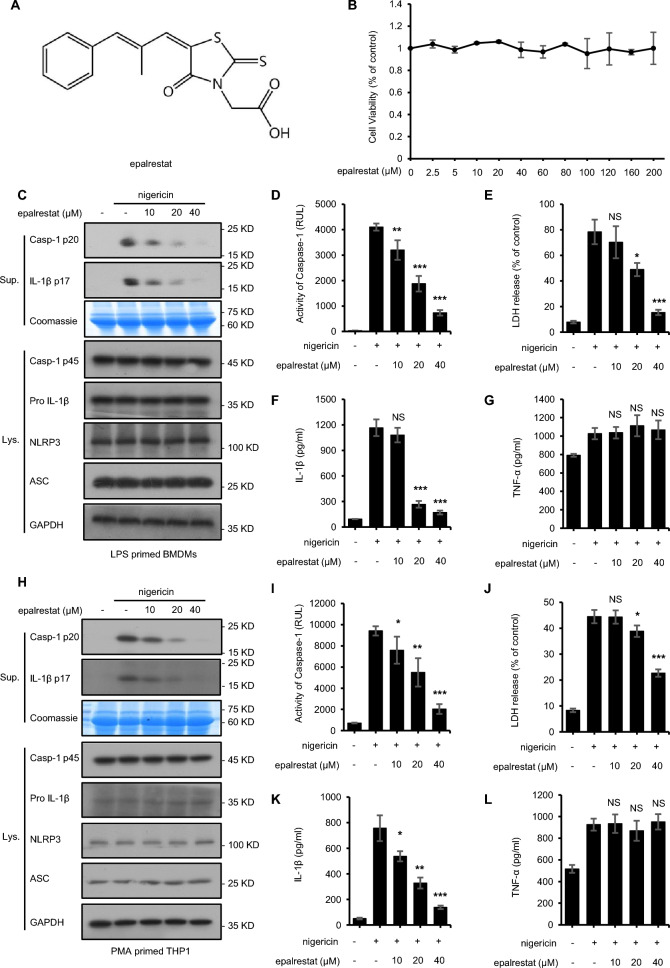
Epalrestat inhibits NLRP3 inflammasome activation in BMDMs and THP1 cells. **A** The structure of epalrestat. **B** Cell viability of BMDMs treated with epalrestat for 24 h as detected using a CCK-8 reagent. **C** LPS-primed BMDMs were treated with various doses of epalrestat for 1 h before stimulation with nigericin for 30 min. Immunoblot analysis of epalrestat was used to detect cleaved caspase-1 and IL-1β in the cell supernatants (Sup.) and the expression of NLRP3, caspase-1 p45, pro-IL-1β, and ASC in the cell lysates (Lys.). **D–G** The activity of caspase-1 **D**, secretion of IL-1β **E**, release of LDH **F**, and the production of TNF-α **G** in the Sup were assessed from samples described in **C**. **H** PMA-primed THP1 cells were treated with various doses of epalrestat for 1 h before stimulation with nigericin for 30 min. Immunoblot analysis of epalrestat was used to detect cleaved caspase-1 and IL-1β in the Sup and the expression of NLRP3, caspase-1 p45, pro-IL-1β, and ASC in the Lys. **I–L** The activity of caspase-1 **I**, secretion of IL-1β **J**, release of LDH **K**, and the production of TNF-α **L** in the Sup were assessed from samples described in **H**. Data are presented as the mean ± SD from at least three biological samples. Statistical differences were analyzed using an unpaired Student’s *t*-test. *P < 0.05, **P < 0.01, ***P < 0.001; *ns* not significant

Bioluminescence assays used for high-throughput screening showed that epalrestat might be a potential inhibitor of NLRP3 inflammasomes (Fig. 1A). As a further investigation of epalrestat's effects on the activation of the NLRP3 inflammasome, we determined its cytotoxicity in BMDMs. We found that epalrestat is not cytotoxic to BMDMs, even at concentrations up to 200 μmol/L (Fig. 1B). Studies have shown that activation of the NLRP3 inflammasome leads to the maturation and secretion of large amounts of pro-caspase-1 and pro-IL-1β [41, 42]. The results showed that epalrestat significantly inhibited caspase-1 activation, IL-1β maturation, and LDH release (Fig. 1C–G, Additional file 1: Fig. S1). Contrary to IL-1β, epalrestat has no effect on the secretion of TNF-α, an inflammasome-independent cytokine (Fig. 1C–G, Additional file 1) [18]. Similarly, epalrestat also significantly inhibited the production of caspase-1 and IL-1β and the nigericin-stimulated release of LDH in THP1 cells, but TNF-α expression was not affected (Fig. 1H–L). We also evaluated the expression of NLRP3 inflammasome complex proteins, including NLRP3, pro-IL-1β, caspase-1 p45, and ASC in the cell lysates. The results showed that epalrestat did not affect the expression of NLRP3 inflammasome complex proteins (Fig. 1C–L, Additional file 1: Fig. S1). Additionally, we compared the efficacy of epalrestat and other NLRP3 inhibitors, including sulforaphane, parthenolide and OLT1177. The results showed that epalrestat, sulforaphane, parthenolide and OLT1177 could inhibit the activation of NLRP3 inflammasome at the concentration of 40 μmol/L, and epalrestat is more effective than OLT1177 at this concentration (Additional file 2). These results confirm that epalrestat inhibits NLRP3 inflammasome activation in LPS-primed BMDMs and THP1 cells in vitro.

### Epalrestat inhibits canonical and noncanonical NLRP3 inflammasome activation but does not affect NLRC4 and AIM2 inflammasome activation

**Fig. 2:**
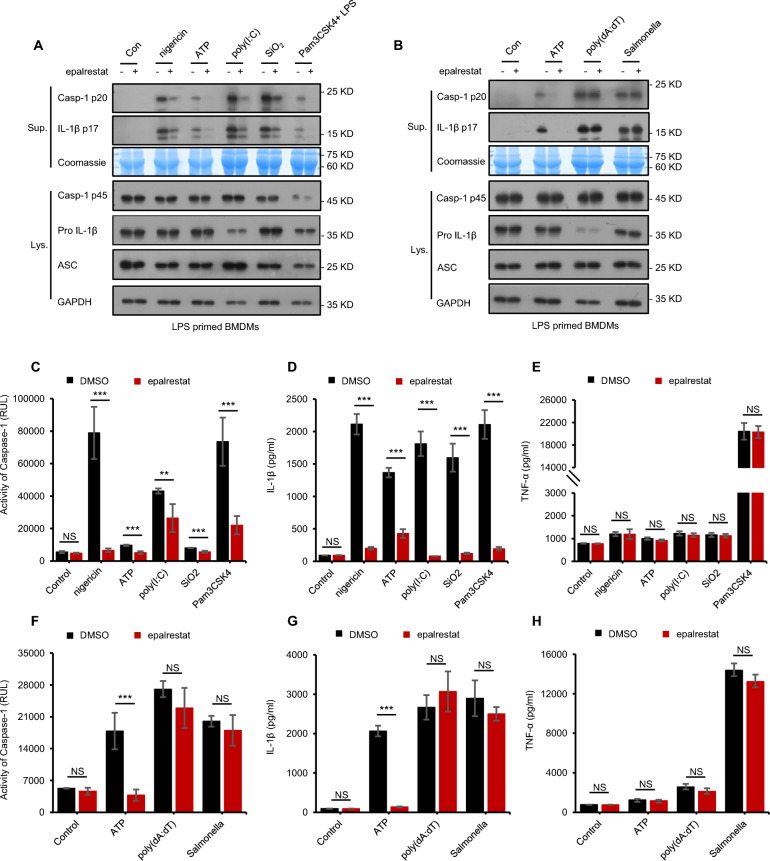
Epalrestat inhibits canonical and noncanonical NLRP3 inflammasome activation but has no effect on NLRC4 and AIM2 inflammasome activation. **A** LPS-primed BMDMs were treated with or without epalrestat (40 μM) before stimulation with ATP, nigericin, SiO_2_, or poly(I: C) and Pam3CSK4-primed BMDMs were treated with or without epalrestat (40 μM) before stimulation with LPS. Immunoblot analysis of epalrestat was used to detect the cleaved caspase-1 and production of IL-1β in the Sup and the expression of caspase-1 p45, pro-IL-1β, and ASC in the Lys. **B** LPS-primed BMDMs were treated with or without epalrestat (40 μM) before stimulation with ATP, poly (dA: dT), or *Salmonella*. Immunoblot analysis of epalrestat was used to detect cleaved caspase-1 and IL-1β in the Sup and the expression of caspase-1 p45, pro-IL-1β, and ASC in the Lys. **C–H** The activity of caspase-1 **C**, **F**, secretion of IL-1β **D**, **G**, and the production of TNF-α **E**, **H** in the Sup were assessed from samples described in **A** or **B**. Data are presented as the mean ± SD from at least three biological samples. Statistical differences were analyzed using an unpaired Student’s *t*-test. *P < 0.05, **P < 0.01, ***P < 0.001; ns, not significant

Pathogen-associated molecular patterns (PAMPs) and danger-associated molecular patterns (DAMPs) could activate the NLRP3 inflammasome, which involve nigericin, SiO2, MSU, ATP, and poly (I: C) [22, 43, 44]. Therefore, we investigated the role of epalrestat in NLRP3 inflammasome activation induced by other stimuli. Epalrestat inhibited the production of caspase-1 and IL-1β triggered by ATP, nigericin, SiO_2_, and poly (I: C) in BMDMs (Fig. 2A, C–E). In accordance with previous studies, epalrestat did not affect the expression of NLRP3 inflammasome complex proteins in cell lysates or TNF-α in cell supernatants (Fig. 2A, C–E). These results indicate that epalrestat could inhibit NLRP3 inflammasome activation mediated by multiple agonists.

Caspase-11 can be activated by intracellular LPS or gram-negative bacteria to target noncanonical NLRP3 inflammasome activation. Therefore, the effect of epalrestat on the noncanonical NLRP3 inflammasome activation was test. As expected, epalrestat could suppress caspase-11-dependent caspase-1 cleavage and IL-1β secretion triggered by LPS after treatment in Pam3CSK4-primed BMDMs (Fig. 2A, C–E). Furthermore, epalrestat did not affect the levels of TNF-α in the cell supernatants or NLRP3 expression in the cell lysates (Fig. 2A, C–E). In conclusion, epalrestat, a broad NLRP3 inflammasome inhibitor, inhibited canonical and noncanonical NLRP3 inflammasome activation.

To further investigate that epalrestat is a specific NLRP3 inflammasome inhibitor, we evaluated its role in NLRC4 inflammasome and AIM2 inflammasome activation. The NLRC4 inflammasome can be activated by flagellin derived from bacteria, including *Salmonella typhimurium*, and the AIM2 inflammasome can be activated by double-stranded DNA [45, 46]. We verified that epalrestat does not affect NLRC4- or AIM2-dependent caspase-1 cleavage and the secretion of IL-1β from *Salmonella typhimurium* or poly (dA:dT) stimulation (Fig. 2B, F–H). Consistent with previous results, the expression of inflammasome complex proteins, including caspase-1 p45, pro-IL-1β, and ASC, and the secretion of TNF-α were not affected by epalrestat (Fig. 2B, F–H). Thus, these results showed that epalrestat was not affected the activation of NLRC4 and AIM2 inflammasomes.

**Fig. 3:**
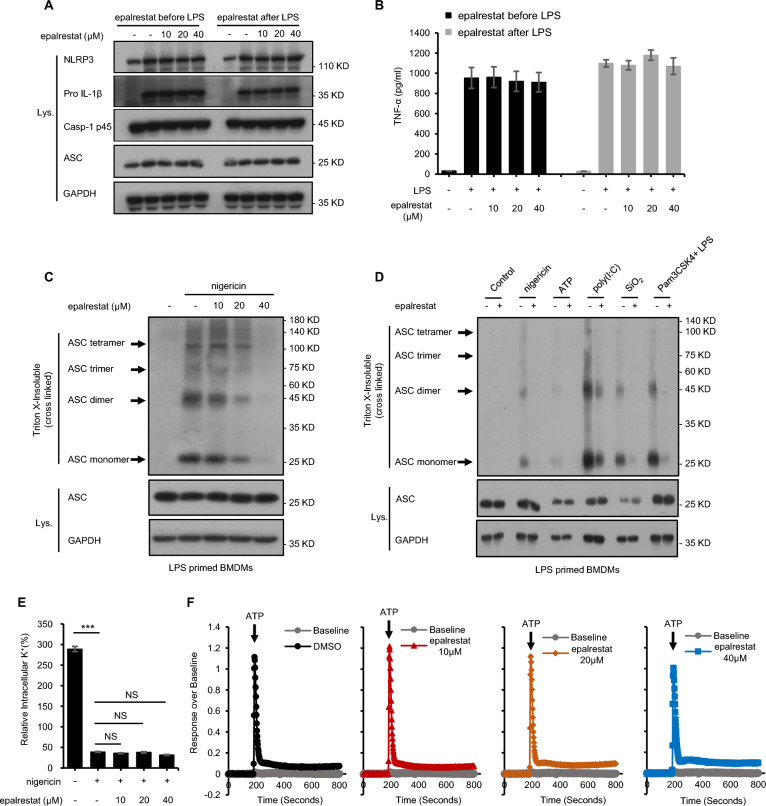
Epalrestat blocks ASC oligomerization but does not affect K^+^ or Ca^2+^ efflux. **A** Immunoblot analysis of BMDMs treated with epalrestat for 1 h before LPS stimulation for 4 h or BMDMs treated with epalrestat for 1 h after LPS stimulation for 1 h. **B** ELISA of TNF-α from samples described in **A**. **C–D** LPS-primed BMDMs were treated with epalrestat (40 μM) before stimulation with ATP, nigericin, SiO_2_, or poly (I: C) and Pam3CSK4-primed BMDMs were treated with epalrestat (40 μM) before stimulation with LPS. Immunoblot analysis of epalrestat was used to detect cross-linked ASC in the Triton X-insoluble pellet. **E** Quantification of intracellular potassium levels in LPS-primed BMDMs pre-treated with various doses of epalrestat and stimulated with nigericin. **F** A trace showing ATP-induced Ca^2+^ flux was measured using a FLIPRT Tetra system in LPS-primed BMDMs treated with epalrestat. Data are presented as the mean ± SD from at least three biological samples. Statistical differences were analyzed using an unpaired Student’s *t*-test. *P < 0.05, **P < 0.01, ***P < 0.001; *ns* not significant

Previous studies have shown that NLRP3 inflammasome activation in macrophages requires a two-step process, with the first phase (priming) being provided by microbes or endogenous molecules that can activate NF-kB to induce the expression of NLRP3 and pro-IL-1β, and the second phase (activation) being triggered by ATP, nigericin or MSU [43, 47–49]. Meanwhile, other studies have shown that unlike ASC and caspase-1, the protein amounts of NLRP3 in resting macrophages are thought to be insufficient for NLRP3 activation [43, 47, 50, 51]. Next, we examined the effect of epalrestat on the expression of NF-κB-dependent NLRP3 and pro-IL-1β. The results suggested that epalrestat did not affect LPS-induced NLRP3 and pro-IL-1β expression when BMDMs were stimulated with epalrestat for 1 h after being pre-treated with LPS for 4 h or stimulated with epalrestat for 1 h before LPS treated for 4 h (Fig. 3A). Similarly, we also detect the production of TNF-α, and the results showed that epalrestat does not affect TNF-a production in these conditions (Fig. 3B). Thus, these results indicated that epalrestat does not suppress the NF-κB-dependent expression of NLRP3 or pro IL-1β and have no effect on the priming stage on the NLRP3 inflammasome activation.

### Epalrestat blocks ASC oligomerization but does not affect K^+^ and Ca^2+^ efflux

As a key step, the adaptive protein ASC forms a single large perinuclear focus per cell during inflammasome activation [52–54]. Therefore, we investigated whether ASC oligomerization is a key target in NLRP3 inflammasome activation during epalrestat treatment. Cytosolic fractions were cross-linked, which from cell lysates, and then visualized via immunoblotting to detect ASC monomers and higher-order complexes. Our results revealed that epalrestat could dose-dependently inhibit ASC oligomerization after stimulated with nigericin (Fig. 3C). The effect of other NLRP3 inflammasome stimulis in the presence or absence of epalrestat also was detected and found that ASC oligomerization could be suppressed upon multiple agonists-mediated NLRP3 inflammasome activation in LPS-primed BMDMs during epalrestat treatment (Fig. 3D). Consistent with previous results, ASC oligomerization induced by *Salmonella typhimurium* or poly (dA:dT) was not influenced by epalrestat treatment (Additional file 3). These results provide robust evidence that epalrestat does not affect ASC oligomerization during NLRC4 and AIM2 inflammasome activation. Moreover, the effect of epalrestat on the NLRP3 inflammasome activation is independent of ASC oligomerization, so we further investigate the upstream process of NLRP3 inflammasome activation to search for the mechanism by which epalrestat inhibits NLRP3 inflammasome activation.

We then investigated the mechanisms involved in NLRP3 inflammasome activation by epalrestat, such as K^+^ efflux, Ca^2+^ mobilization, and ROS generation [54–56]. We first evaluated the effect of epalrestat on K^+^ efflux. The intracellular potassium levels could significantly decrease upon stimulation with nigericin. However, epalrestat does not influence this effect; this indicates that epalrestat inhibits NLRP3 inflammasome activation but not potassium efflux (Fig. 3E). In addition to NLRP3 inflammasome activation, flow cytometry was used to analyze ROS production. We did not observe a change in the ROS generated in BMDMs pre-treated with epalrestat and stimulated by nigericin, poly (dA:dT), or *Salmonella typhimurium* (Additional file 4). The results of the Ca^2+^ mobilization assay upon pre-treatment with epalrestat and stimulation with ATP also showed that epalrestat does not affect Ca^2+^ mobilization upstream of NLRP3 activation (Fig. 3F). Thus, NLRP3 activation is therefore postulated to be controlled by epalrestat acting downstream of K + efflux, Ca2 + flux, and ROS production.

### Aldose reductase is an important target in epalrestat-induced NLRP3 inflammasome activation

**Fig. 4:**
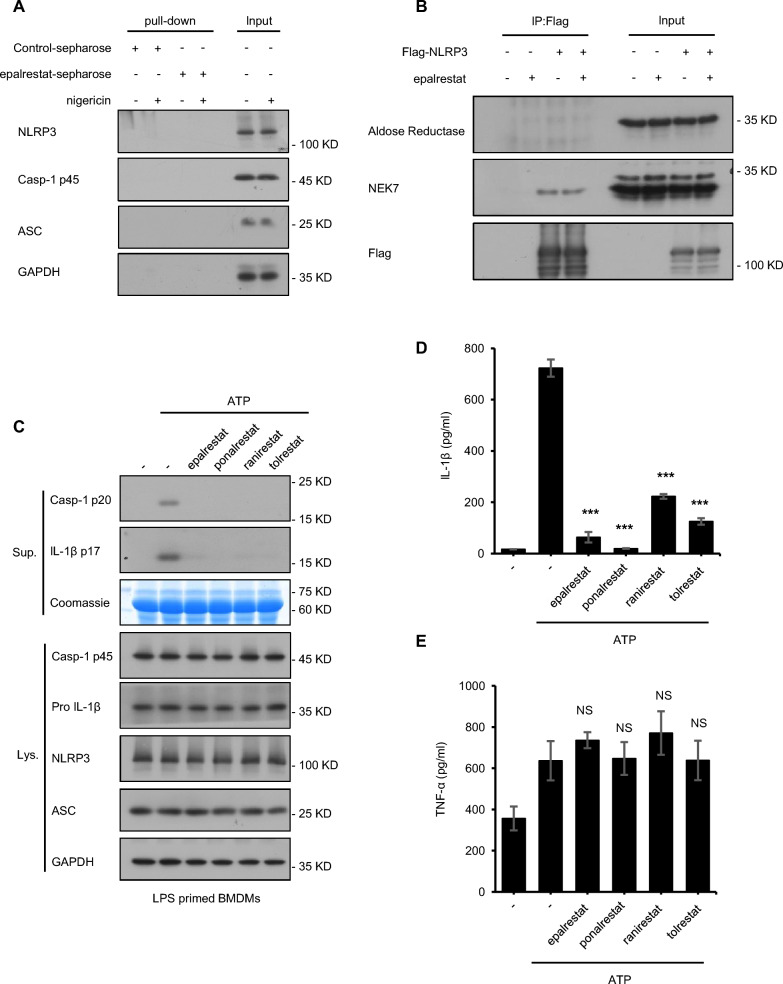
Aldose reductase is a key target in NLRP3 inflammasome activation during epalrestat treatment.** A** Cell lysates of LPS-primed BMDMs stimulated with or without nigericin. Cells were incubated with epalrestat-sepharose for 12 h and the proteins were pulled down with Sepharose beads. **B** HEK-293 T cells were transfected with Flag-Vector or Flag-NLRP3 for 24 h and then treated with or without epalrestat. Immunoprecipitation was performed using anti-Flag M2 agarose beads. The results of the immunoblot analysis for Flag and NEK7 are shown. **C** LPS-primed BMDMs were treated with epalrestat, ponalrestat, ranirestat, and tolrestat for 1 h and then stimulated with ATP. Immunoblot analysis was used to detect cleaved caspase-1 and IL-1β in the Sup and the expression of NLRP3, caspase-1 p45, pro-IL-1β, and ASC in the Lys. **D-E** ELISA of IL-1β **D** and TNF-α **E** in the Sup was assessed from samples described in **C**. Data are presented as the mean ± SD from at least three biological samples. Statistical differences were analyzed using an unpaired Student’s *t*-test. *P < 0.05, **P < 0.01, ***P < 0.001; *ns* not significant

To further elucidate whether epalrestat directly binds to the protein responsible for activating the NLRP3 inflammasome, such as NLRP3, caspase-1, NEK7, and ASC, epalrestat was conjugated with EAH-activated Sepharose (epalrestat-sepharose) and the control group was conjugated with control Sepharose (epalrestat-control). Epalrestat-interacting proteins were then subjected to a pull-down assay from the cell lysates for detection. And the results revealed that ASC, NLRP3, and caspase-1 were not pulled down by epalrestat-sepharose (Fig. 4A). Previous studies have demonstrated that epalrestat is a specific aldose reductase inhibitor. Thus, we detected whether aldose reductase could be pulled down by epalrestat-sepharose; the results showed that aldose reductase was not pulled down by epalrestat-sepharose. Next, we investigated whether aldose reductase interacts with NLRP3 during the activation of inflammasome. Flag-tagged NLRP3, and a semi-endogenous co-immunoprecipitation experiment were transfected into HEK-293 T cells using anti-Flag M2 beads. The co-immunoprecipitation experiment results showed that aldose reductase does not interact with NLRP3 upon epalrestat treatment (Fig. 4B). Conversely, NEK7 interacts with NLRP3 in HEK-293 T cells, promoting NLRP3 oligomerization and ASC recruitment to NLRP3; this interaction was not influenced by epalrestat treatment. Therefore, the mechanism underlying the effect of epalrestat on NLRP3 inflammasome activation does not involve the interaction between NLRP3 and aldose reductase.

To elucidate the mechanism of epalrestat on inhibiting NLRP3 inflammasome activation, we also tested whether aldose reductase is a critical target of NLRP3 inflammasome activation. Previous studies have reported that epalrestat, ponalrestat, ranirestat, and tolrestat are all aldose reductase inhibitors [57]. Thus, we evaluated their effects on NLRP3 inflammasome activation. Consistent with our expectation, the activation of caspase-1 and the maturation of IL-1β could be inhibited by epalrestat, ponalrestat, ranirestat, and tolrestat but not TNF-α, suggesting that aldose reductase inhibitors could suppress NLRP3 inflammasome activation (Fig. 4C–E). Taken together, aldose reductase is a key target of epalrestat to inhibit NLRP3 inflammasome activation.

### Epalrestat ameliorates LPS-induced NLRP3 inflammasome activation in vivo

**Fig. 5:**
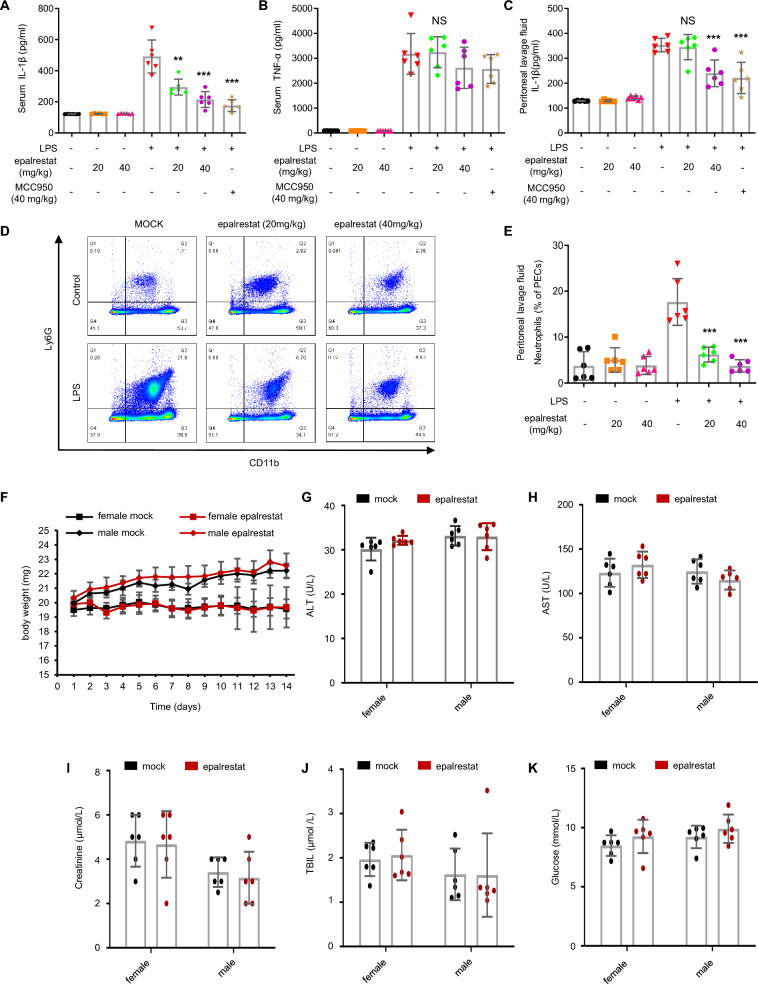
Epalrestat ameliorates LPS-induced NLRP3 inflammasome activation in vivo*.* **A–E** Mice were intraperitoneally injected with LPS or PBS for 6 h after pre-treatment with epalrestat or vehicle. ELISA of IL-1β **A** and TNF-α **B** in the serum and IL-1β **C** in the peritoneal lavage fluid was performed. Flow cytometric analysis of peritoneal cell exudates **D**–**E**. **F-K** Male or female mice were gavaged with epalrestat (120 mg/kg/day) or vehicle daily for 14 days. Changes in the body weight **F**, and AST **G**, ALT **H**, creatinine **I**, TBIL **J**, and glucose **K** levels were measured. Data are presented as the mean ± SD. Statistical differences were analyzed using an unpaired Student’s *t*-test. *P < 0.05, **P < 0.01, ***P < 0.001; *ns* not significant

To assess the effect of epalrestat in vivo, we employed NLRP3-dependent inflammatory models using LPS. Previous studies have reported that LPS injection in mice induced NLRP3-dependent IL-1β production and recruitment of inflammatory cells. Here, mice were intraperitoneally injected with LPS for 4 h after treatment with epalrestat or MCC950 for 1 h. Afterwards, serum and peritoneal lavage fluid were gathered to measure the concentration of inflammasome-independent cellular factors. The data showed that epalrestat and MCC950 dose-dependently suppressed IL-1β and TNF-α production (Fig. 5A–C). Similarly, epalrestat and MCC950 could inhibit LPS- triggered IL-1β expression in peritoneal lavage cells. Similar to the suppressive role of epalrestat on pro-inflammatory cytokines, peritoneal macrophages pre-treated with epalrestat could also be decrease, as measured via flow cytometry (Fig. 5D–E). These results suggest that epalrestat inhibits LPS-induced cytokine release through NLRP3 inflammasome activation in mice. Additionally, safety of epalrestat, which is currently available for clinical use in many countries, was also evaluated in vivo. Briefly, mice were gavaged daily for 14 days with epalrestat (120 mg/kg) at a three-fold higher dose than the one used for the LPS-triggered NLRP3 inflammasome activation experiments. We found that biochemical parameters, including plasma ALT, AST, creatinine, TBIL, and glucose, as well as body weight, were not influenced by epalrestat treatment, indicating that epalrestat is well-tolerated and safe in vivo (Fig. 5F–K).

### Epalrestat exhibits a therapeutic effect in a NASH mouse model

**Fig. 6:**
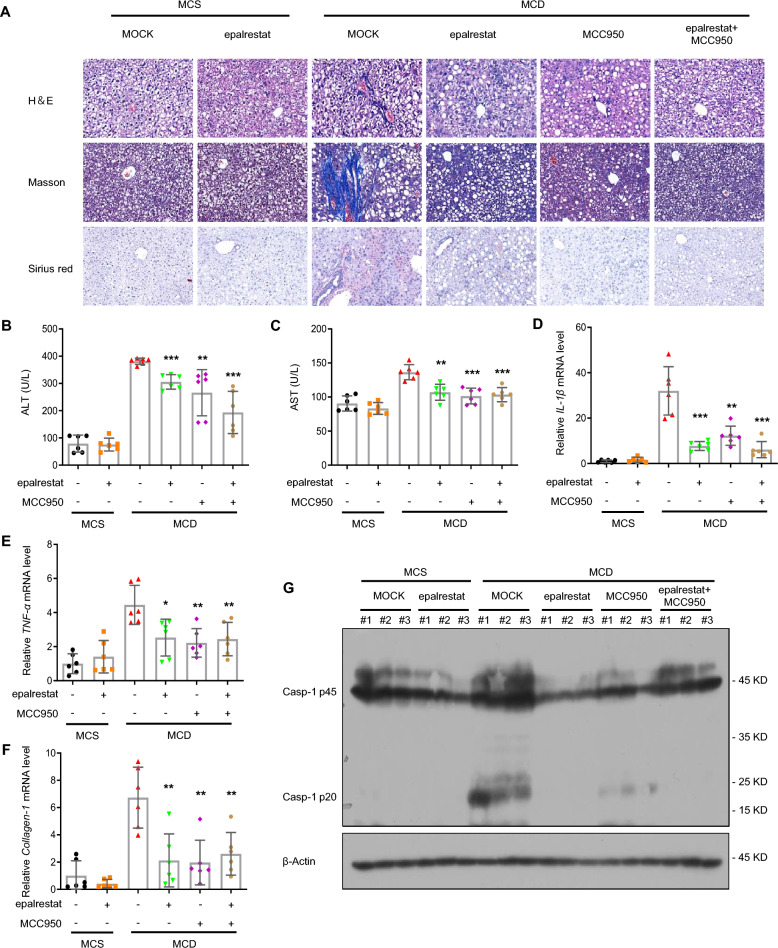
Epalrestat exhibits a therapeutic effect in a NASH mouse model. Male mice were fed with an MCD or MCS diet for 6 weeks. The mice were then randomly separated into groups and gavaged with epalrestat (20 mg/kg), MCC950 (20 mg/kg), or vehicle daily for a total of 5 days and then with 40 mg/kg every other day, for up to 6 weeks. **A** Macroscopic appearance of the liver and representative micrographs of liver sections stained with H&E, Masson stain, and Sirius red. Scale bars represent 100 μm. **B–C** Serum levels of ALT **B** and AST **C**. **D–F** *IL-1β* **D** *TNF-α* **E** and *Colla1* **F** mRNA levels were measured from the liver tissue. **G** The levels of pro-caspase-1, and cleaved caspase-1 proteins in the liver tissue were determined using immunoblot analysis. Data are presented as the mean ± SD. Statistical differences were analyzed using an unpaired Student’s *t*-test. *P < 0.05, **P < 0.01, ***P < 0.001; *ns* not significant

Previous studies have reported that epalrestat is used to modify peripheral nerve function in diabetic patients, so we investigated whether new indications, such as for NASH, could be added. As reported, NASH could be caused by an MCD diet and the inflammatory response, and regulated by the sustained NLRP3 inflammasome activation. To estimate the influence of epalrestat on NASH, MCD or MCS diets were fed to mice to generate a NASH mouse model that can exhibit a few characteristics of NASH, including hepatocyte ballooning, hepatic steatosis, inflammatory cell infiltration of the liver lobules, and fibrosis. We observed that compared to MCS diet-treated mice, hepatic steatosis and fibrosis, and inflammatory factor infiltration were discovered in the MCD diet-treated mice. However, these pathological changes were significantly reduced after epalrestat treatment, as well as MCC950, an NLRP3 inflammasome inhibitor, when used as a positive control (Fig. 6A). Furthermore, the expression of ALT and AST in plasma, which were increased after MCD diet, were also alleviated by epalrestat or MCC950 treatment. Interestingly, treatment with a combination of epalrestat and MCC950 yielded similar effects to treatment with epalrestat or MCC950 alone in MCD diet-triggered NASH (Fig. 6B–C).

Next, we clarify whether epalrestat treatment is achieved by inhibiting NLRP3 inflammasome activation in NASH, so active caspase-1 expression and pro-inflammatory cytokine secretion were tested in liver tissue after epalrestat treatment. Consistent with the previous results, *IL-1β* and *TNF-α* mRNA expression, pro-inflammatory cytokine genes, were markedly decreased upon treatment with epalrestat or MCC950 (Fig. 6D, E). As expected, caspase-1 p20 expression in the liver tissue of MCD diet-treated mice was significantly reduced upon epalrestat or MCC950 treatment (Fig. 6G). In summary, we evaluated the mRNA transcription of genes involved in hepatic fibrogenesis. The mRNA levels of collagen 1 in the liver were reduced by epalrestat or MCC950 treatment (Fig. 6F). The results of the combination of epalrestat and MCC950 treatment on NASH also showed that caspase-1 p20 expression, and *IL-1β* and *TNF-α* mRNA levels in the liver tissue could be suppressed by the combination treatment and the therapeutic effect better than both administered separately (Fig. 6D–G). In conclusion, our results suggest that epalrestat alleviates liver inflammation and pathology in NASH by inhibiting NLRP3 inflammasome activation.

## Discussion

Our study revealed that epalrestat has a powerful suppressive function on NLRP3 inflammasome activation. As a powerful and specific NLRP3 inhibitor, epalrestat is effective in vitro in BMDMs and in vivo in mice. We also revealed that epalrestat has a potential therapeutic in NLRP3 inflammasome-driven diseases, such as NASH. Until now, there only a few small molecules have been reported to directly target the NLRP3 inflammasome, but OLT1177 is the only one that has undergone phase II clinical trials [28]. In this research, we describe the effect of epalrestat, a clinically effective and safe drug that could also improve the function of peripheral nerves in diabetes mellitus. Therefore, epalrestat may be useful and important for treating NLRP3-driven diseases.

We also elucidated the mechanism by which epalrestat inhibits NLRP3 inflammasome in this study. The data indicated that treatment with epalrestat, either before or after LPS stimulation, failed to inhibit NF-κB-mediated pro-IL-1β expression and TNF-α production. Moreover, epalrestat has no effect on K^+^ efflux, Ca^2+^ flux, or ROS production but could dose-dependently block ASC oligomerization. We also characterized the interaction between epalrestat and inflammasome-associated proteins and indicated that epalrestat could not directly interact with the proteins essential for NLRP3-inflammasome activation, such as NLRP3, caspase-1, NEK7, and ASC.

Epalrestat, as an aldose reductase inhibitor, could improve the function of peripheral nerves in diabetes patients and has been successfully used in the clinic [34, 39, 40]. Aldose reductase is a key rate-limiting enzyme of the polyol pathway, and controlling its activity can lower blood glucose and thus alleviate diabetic complications [36, 58–60]. However, recent research evidence suggests that aldose reductase is an outstanding facilitator to regulate inflammatory signals [37, 61]. Accordingly, preventing inflammatory complications may be a potential use of aldose reductase inhibition. Currently, aldose reductase inhibitors are used to treat endotoxemia, sepsis, or inflammatory diseases [62–64]. Aldose reductase inhibitors have been clinically studied for diabetic complications over the past few years [62, 65]. Therefore, aldose reductase inhibitors could be explored as a treatment for inflammatory diseases. Besides epalrestat, ponalrestat, ranirestat, and tolrestat were also selected in this study to verify whether aldose reductase inhibitors could block NLRP3 inflammasome activation. However, epalrestat is the only aldose reductase inhibitor that is effective and safe in clinical use [34, 66]. We have demonstrated that epalrestat could suppress NLRP3 inflammasome activation by targeting aldose reductase.

NASH is characterized by hepatocyte ballooning, hepatic steatosis, inflammatory cell infiltration of the liver lobules, hepatocellular injury and fibrosis [67, 68]. Several studies indicate that NLRP3 inflammasomes could induce tissue damage and liver fibrosis in NASH [20, 69, 70]. In our study, epalrestat could target NLRP3 inflammasome to alleviate liver inflammation and fibrosis in MCD-fed mice and has comparable therapeutic potential to MCC950, which may be a plausible direction for NASH pharmacotherapy. Therefore, another important discovery is that epalrestat could decrease liver inflammation and fibrosis by suppressing NLRP3 inflammasome activation in NASH.

In this research, we identified epalrestat as a prospective novel and potential powerful NLRP3 inflammasome antagonist. Epalrestat inhibited NLRP3 inflammasome activation in vitro and in vivo through its function of inhibiting aldose reductase activity. Epalrestat also displayed significant therapeutic potential in NASH. Unfortunately, there are still no FDA-approved medical treatments available for NASH. Epalrestat is a clinically effective and safe drug and has already been used in many countries, such as China, Japan, and India; therefore, epalrestat may be used as a favorable candidate drug for treating NASH.

### Supplementary Information

**Additional file 1:** Epalrestat inhibits NLRP3 inflammasome activation in BMDMs triggered by ATP.

**Additional file 2:** Epalrestat, sulforaphane, parthenolide and OLT1177 can inhibit NLRP3 inflammasome activation in BMDMs triggered by ATP.

**Additional file 3:** Epalrestat have no effect on the ASC oligomerization on the activation of NLRC4 and AIM2 inflammasome.

**Additional file 4:** Epalrestat do not affect the production of ROS.

## Data Availability

Not applicable.
