# Supplementary material for: Novel role for epalrestat: protecting against NLRP3 inflammasome-driven NASH by targeting aldose reductase
Source: J Transl Med. 2023 Oct 7;21:700. doi: 10.1186/s12967-023-04380-4 (PMC10560438; doi:10.1186/s12967-023-04380-4)
Supplement: Supplementary file 1 — Additional file 1: Epalrestat inhibits NLRP3 inflammasome activation in BMDMs triggered by ATP. [file 12967_2023_4380_MOESM1_ESM.docx]

**
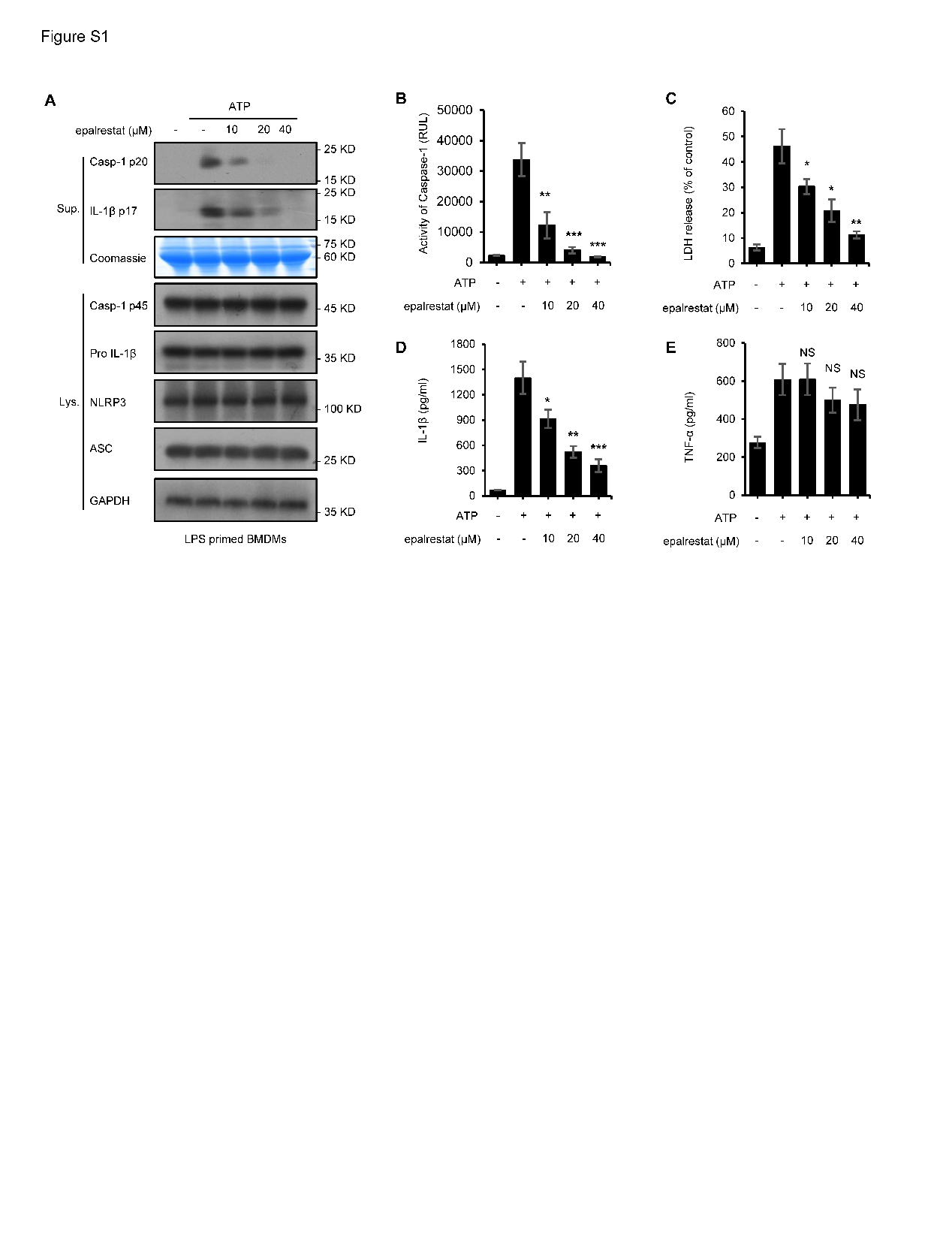
**

**Epalrestat inhibits NLRP3 inflammasome activation in BMDMs** **triggered by ATP** LPS-primed BMDMs were treated with various doses of epalrestat for 1h before stimulate with ATP. **A** Immunoblot analysis of epalrestat were used to detected the cleaved caspase-1 and production of IL-1β in cell sup. and the expression of NLRP3, caspase-1 p45, pro IL-1β and ASC in Lys.. **B-E** Activity of caspase-1 (B), secretion of IL-1β (C), the release of LDH (D) and the production of TNF-α (E) in Sup. were assessed. Data are presented as mean±SD from at least three biological samples. Statistics differences were analyzed using an unpaired Student's t-test: *P < 0.05, **P < 0.01, ***P < 0.001; ns, not significant.
