# Supplementary material for: Novel role for epalrestat: protecting against NLRP3 inflammasome-driven NASH by targeting aldose reductase
Source: J Transl Med. 2023 Oct 7;21:700. doi: 10.1186/s12967-023-04380-4 (PMC10560438; doi:10.1186/s12967-023-04380-4)
Supplement: Supplementary file 2 — Additional file 2: Epalrestat, sulforaphane, parthenolide and OLT1177 can inhibit NLRP3 inflammasome activation in BMDMs triggered by ATP. [file 12967_2023_4380_MOESM2_ESM.docx]

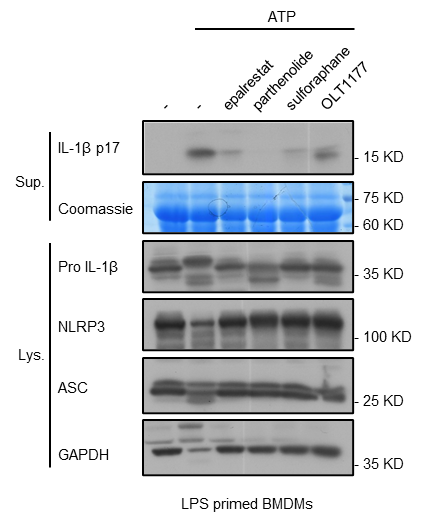


**Epalrestat, sulforaphane, parthenolide and OLT1177 can inhibit NLRP3 inflammasome activation in BMDMs triggered by ATP** LPS-primed BMDMs were treated with epalrestat, sulforaphane, parthenolide or OLT1177 for 1h before stimulate with ATP. Immunoblot analysis of epalrestat, sulforaphane, parthenolide and OLT1177 were used to detected the production of IL-1β in cell sup. and the expression of NLRP3, pro IL-1β and ASC in Lys.. were assessed.
