# Supplementary material for: Novel role for epalrestat: protecting against NLRP3 inflammasome-driven NASH by targeting aldose reductase
Source: J Transl Med. 2023 Oct 7;21:700. doi: 10.1186/s12967-023-04380-4 (PMC10560438; doi:10.1186/s12967-023-04380-4)
Supplement: Supplementary file 3 — Additional file 3: Epalrestat have no effect on the ASC oligomerization on the activation of NLRC4 and AIM2 inflammasome. [file 12967_2023_4380_MOESM3_ESM.docx]

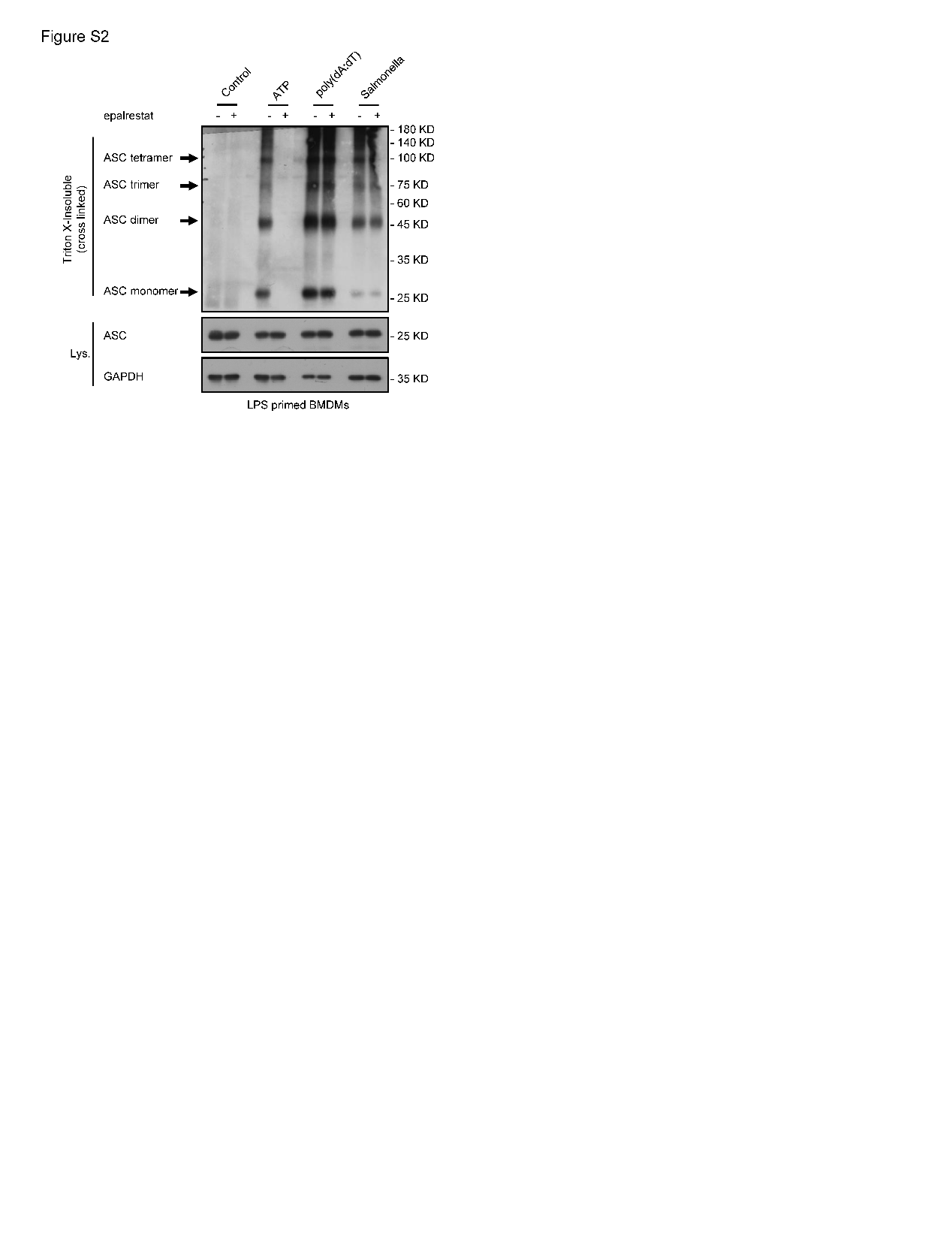


**Epalrestat have no effect on the ASC oligomerization on the activation of NLRC4 and AIM2 inflammasome** LPS-primed BMDMs were treated with epalrestat (40μM) before stimulate with ATP, poly (dA:dT) or *salmonella*. Immunoblot analysis of epalrestat were used to detected the cross-linked ASC in the Triton X-insoluble pellet. Immunoblot analysis of epalrestat were used to detected the cleaved caspase-1 and production of IL-1β in cell sup. and the expression of caspase-1 p45, pro IL-1β and ASC in Lys..
