# Supplementary material for: Novel role for epalrestat: protecting against NLRP3 inflammasome-driven NASH by targeting aldose reductase
Source: J Transl Med. 2023 Oct 7;21:700. doi: 10.1186/s12967-023-04380-4 (PMC10560438; doi:10.1186/s12967-023-04380-4)
Supplement: Supplementary file 4 — Additional file 4: Epalrestat do not affect the production of ROS. [file 12967_2023_4380_MOESM4_ESM.docx]

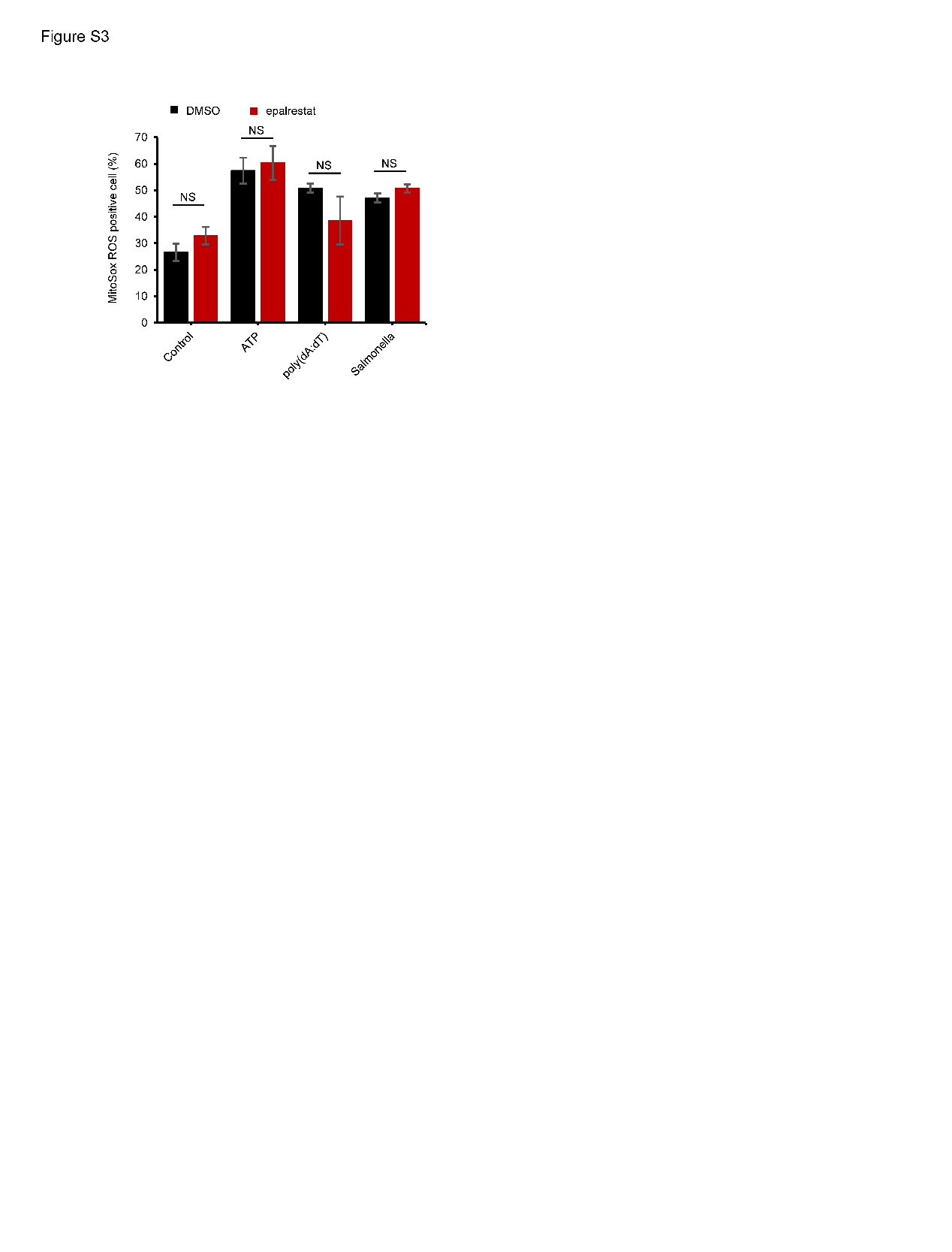


**Epalrestat do not affect the production of ROS** Percentage of ROS-positive cells in LPS-primed BMDMs with epalrestat and then stimulated with ATP, poly (dA:dT) or *salmonella*, followed by staining with MitoSox. Data are presented as mean±SD from at least three biological samples. Statistics differences were analyzed using an unpaired Student's t-test: *P < 0.05, **P < 0.01, ***P < 0.001; ns, not significant.
